# Synthesis and Properties of Phosphoric-Acid-Doped Polybenzimidazole with Hyperbranched Cross-Linkers Decorated with Imidazolium Groups as High-Temperature Proton Exchange Membranes

**DOI:** 10.3390/polym12030515

**Published:** 2020-02-27

**Authors:** Chunmei Gao, Meishao Hu, Li Wang, Lei Wang

**Affiliations:** 1 College of Chemistry and Environmental Engineering, Shenzhen University, Shenzhen 518060, China; gaocm@szu.edu.cn; 2 Shenzhen Key Laboratory of Polymer Science and Technology, College of Materials Science and Engineering, Shenzhen University, Shenzhen 518060, China; josundae@hotmail.com (M.H.); 2161120212@email.szu.edu.cn (L.W.)

**Keywords:** polybenzimidazole, fuel cell, proton exchange membrane, high temperature, hyperbranched cross-linker, imidazolium groups

## Abstract

Highly phosphoric-acid (PA)-doped polybenzimidazole (PBI) membranes exhibit good proton conductivity at high temperatures; however, they suffer from reduced mechanical properties and loss of PA molecules due to the plasticity of PA and the weak interactions between PA and benzimidazoles, especially with the absorption of water. In this work, a series of PBIs with hyperbranched cross-linkers decorated with imidazolium groups (ImOPBI-x, where x is the weight ratio of the hyperbranched cross-linker) as high-temperature proton exchange membranes are designed and synthesized for the first time. We observe how the hyperbranched cross-linkers can endow the membranes with improved oxidative stability and acceptable mechanical performance, and imidazolium groups with strong basicity can stabilize the PA molecules by delocalization and hydrogen bond formation to endow the membranes with an enhanced proton conductivity and a decreased loss of PA molecules. We measured a high proton conductivity of the ImOPBI-x membranes, ranging from 0.058 to 0.089 S cm^−1^ at 160 °C. In addition, all the ImOPBI-x membranes displayed good mechanical and oxidative properties. At 160 °C, a fuel cell based on the ImOPBI-5 membrane showed a power density of 638 mW cm^−2^ and good durability under a hydrogen/oxygen atmosphere, indicating its promising use in anhydrous proton exchange membrane applications.

## 1. Introduction

Since proton exchange membrane fuel cells (PEMFCs) can directly convert the chemical energy of hydrogen to electricity with high efficiency and zero emissions, they have been regarded as one of the most promising power sources [1,2,3,4]. Based on their working temperature, PEMFCs can be divided into high-temperature PEMFCs (HT-PEMFCs) and low-temperature PEMFCs (LT-PEMFCs). Due to the distinct advantages of HT-PEMFCs over traditional LT-PEMFCs, such as enhanced kinetics and high catalyst tolerance to CO produced by steam reforming, the development of HT-PEMFCs has recently attracted widespread attention [5,6,7,8]. The core component of PEMFCs is the polymer electrolyte membrane (PEM). In general, PEMs must have sufficient proton conductivity, good mechanical properties, excellent chemical stability, moderate price, and good processability to meet the needs of large-scale preparation. Generally, membranes used in HT-PEMFCs belong to four groups [8]: sulfonated aromatic hydrocarbon polymer membranes [9,10], inorganic–organic composite membranes [11,12,13], membranes of blend polymers [14,15], and acid–base polymer membranes [16,17,18,19], among which phosphoric acid-doped polybenzimidazole (PA/PBI) membranes are regarded as the most promising PEMs for high-temperature operation because of features such as their high-temperature resistance, high proton conductivity, good flexibility and chemical stability, low gas permeability, and low price. [20,21,22].

The proton conductivity of PBI membranes generally increases with the content of PA dopant, which could generate more dynamic hydrogen bond networks beneficial to the proton transfer. However, PBIs with high PA doping will have reduced interaction among PBI chains, resulting in weak mechanical properties which make them unsuitable for practical applications. In addition, the leakage of PA from the PBI matrix due to the weak acid–base interaction between PA and PBI remains a great challenge.

Branched polymers with a unique three-dimensional architecture have better oxidative stability and a larger amount of PA sorption ability than linear polymers; therefore, introducing branching units into PBIs can endow the membranes with better performance. However, they suffer from reduced mechanical properties [23]. Compared to branching, cross-linking is an effective way to improve mechanical strength because it strengthens the interactions among the polymer chains. Recently, cross-linking has been widely explored in PA/PBI membranes for HT-PEMFCs, and improved properties have been achieved compared to those of linear PBI membranes [12,24,25,26,27,28]. Based on the advantages of cross-linking and branching, applying both methods to one membrane can enhance performance. Using 1,3,5-benzene tricarboxylic acid and γ-(2,3-epoxypropoxy)propyltrimethoxysilane (KH560) as the branching and cross-linking agents, respectively, a series of cross-linked poly(aryl ether benzimidazole) derivatives (OPBIs) containing a branching structure (SC-B-OPBI) as membrane materials were successfully prepared for the first time by our group [29]. The PA-doped SC-B-OPBI membranes displayed a significantly improved proton conductivity (0.033–0.044 S cm^−1^ at 180 °C) and tensile strength (11.8–16.9 MPa) compared to the linear membrane (0.022 S cm^−1^ at 180 °C, 11.2 MPa) [29]. In addition, we also developed cross-linked PBIs containing branched structures without sacrificing effective N-H sites by using bis(3-phenyl-3,4-dihydro-2H-1,3benzoxazinyl)isopropane (BA-a) as the cross-linking agent, among which the highest proton conductivity was up to 0.073 S cm^−1^ at 160 °C and the maximum tensile strength at break of 17 MPa [30]. The results indicate that the combination of cross-linking and branching is an effective approach to improve the properties of polybenzimidazole membrane materials; however, their applications are limited because multistep preparation must be used. If one monomer contains both cross-linking and branching groups, a simple route for the synthesis of membranes will be obtained. Recently, a hyperbranched cross-linker (bromomethylated poly(pxylene) (Br-HPP)) was synthesized and introduced to OPBI, which was further reacted with quaternary ammonium (QA) groups, since QA hydroxide has a strong basicity that can cause PA to completely deprotonate by forming cation–biphosphate anion (QA^+^…H_2_PO_4_·(H_3_PO_4_)_n_) interactions [31]. The hyperbranched cross-linked membranes showed a high proton conductivity (0.049 S cm^−1^ at 180 °C), good oxidative resistance, and good mechanical properties (up to 27.0 MPa) [31].

**Scheme 1:**
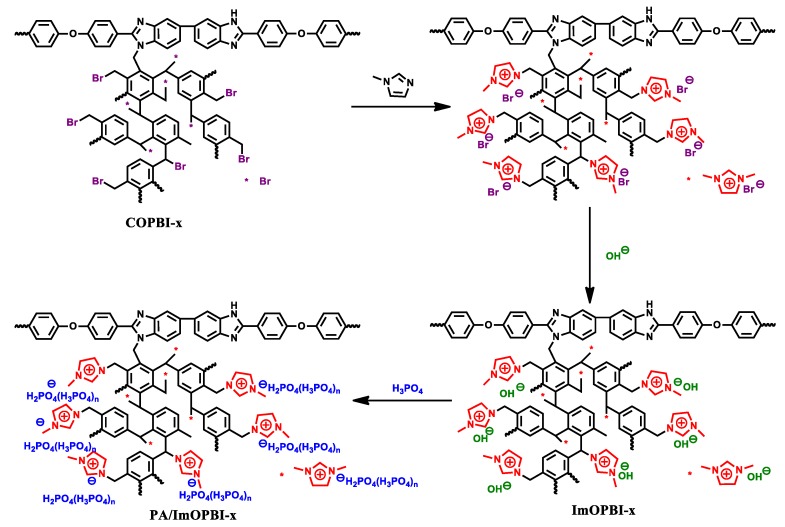
Preparation of polybenzimidazoles (PBIs) with hyperbranched cross-linkers decorated with imidazolium groups (ImOPBI-x, where x is the weight ratio of the hyperbranched cross-linker) and phosphoric-acid-doped ImOPBI-x (PA/ImOPBI-x).

Compared to QA, the imidazolium (Im) group has a conjugated ring structure with a larger space, which can accommodate more PA molecules and stabilize them by delocalization and hydrogen bond formation [17]. A series of polymers containing Im groups have been developed as high-temperature PEMs (HT-PEMs), and the proton conductivity of these membranes was obviously enhanced [16,17,24,32,33]. To further improve performance, this work introduced Ims to the hyperbranched cross-linked OPBI structure (ImOPBI, Scheme 1). The results showed that these membranes exhibited improved proton conductivity compared to QOPBI. We also studied other properties of the ImOPBI-based membranes, including their thermal and oxidative stability, phosphoric acid uptake and retention properties, and mechanical strength, as well as the preliminary results of their fuel cell performances at elevated temperatures.

## 2. Materials and Methods

### 2.1. Preparation of COPBI-x, ImOPBI-x, and OPBI Membranes

The polymer OPBI and hyperbranched cross-linked agents were prepared according to the methods reported in the literature [31]. A calculated amount of OPBI was added to N-methyl pyrrolidone (NMP) to obtain 3 wt% OPBI NMP solution, and then a suitable amount of hyperbranched cross-linked agents were added. The mixture was stirred at room temperature for 12 h, and then it was cast onto the glass plate, dried in a vacuum oven at 80 °C for 3 h, and then slowly heated to 160 °C for 12 h to obtain the COPBI-x membranes.

The COPBI-x membranes were immersed in a 0.5 M 1-methyl imidazole acetone solution for 48 h at 40 °C. After that, the membranes were washed with acetone several times to remove any residual 1-methyl imidazole, and then they were soaked in 0.5 M NaOH for 24 h to convert Br- to OH-. Then, they were washed with water several times to remove the residual alkali solution and dried at 120 °C for 24 h to remove the residual water.

The linear OPBI membranes were prepared by the literature method [31] and were also immersed in 1-methyl imidazole acetone and NaOH solution in turn and then washed by water several times to remove the residual alkali solution and dried at 120 °C for 24 h to remove the residual water.

### 2.2. Characterization Methods

The Fourier-transform infrared (FTIR) spectra of the membranes were recorded using a Nicolet 6700 spectrometer (Nicolet, Waltham, MA, USA). The thermal stabilities of the polymers were measured with a Q50 thermogravimetric analyzer (TA Instruments, New Castle, DE, USA) at a heating rate of 10 °C min^−1^ under an N_2_ flow of 50 mL min^−1^. The mechanical properties of the membranes were evaluated at room temperature on an electromechanical universal test machine (CMT4204, MTS systems, Jinan, China) at a strain rate of 2 mm min^−1^. The Br content of the membranes was determined by X-ray fluorescence spectroscopy (S4 Explorer, Bruker, Karlsruhe, Germany). Typical measurements of the properties of the membranes are described in detail in the supplementary data, including the gel fraction, phosphoric acid uptake, mechanical properties, oxidative stability, and single-cell performance.

## 3. Results and Discussion

### 3.1. Preparation of the ImOPBI-x Membranes

**Figure 1:**
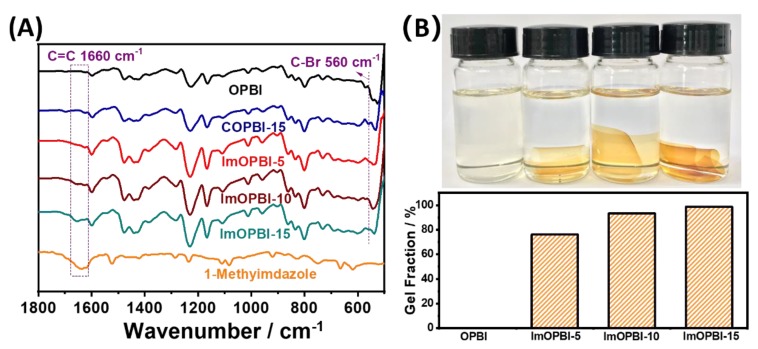
(**A**) FTIR spectra of cross-linked poly(aryl ether benzimidazole) derivatives (OPBI), COPBI-15, ImOPBI-x, and 1-methylimidazole membranes. (**B**) Gel fraction of linear OPBI and ImOPBI membranes.

The preparation of hyperbranched cross-linked OPBI membranes with imidazolium hydroxides (ImOPBI-x, Scheme 1) was similar to that of QOPBI [31]. The S_N_2 alkylation reaction occurred under mild conditions between the cross-linked COPBI and 2-methylimidazole, and the product was then treated with NaOH to achieve ImOPB-x. To prove the successful substitution of imidazolium salts, the infrared spectra of OPBI, COPBI-15, ImOPBI-x, and 1-methylimidazole are compared in Figure 1A. In the spectrum of COPBI-15, the signal at 560 cm^−1^ is a C-Br characteristic peak, indicating the successful introduction of the cross-linking agent. The weakened or lost signals at 560 cm^−1^ in the spectrum of ImOPBI-x indicated that the Br groups had been reacted with 1-methylimidazole or NaOH. In addition, a new band at 1660 cm^−1^ appeared. Compared with the infrared spectrum of 1-methylimidazole, the signals at 1660 cm^−1^ are ascribed to the C=C double bond stretching vibration of the imidazole groups. In the spectra of ImOPBI-5, ImOPBI-10, and ImOPBI-15, the signals of the C=C characteristic peak increased with the increase of cross-linking agents, which proves that the imidazolium salt groups increased with the increase in cross-linking structures.

In addition, cross-linking will decrease the membranes’ solubility. As shown in Figure 1B, OPBI could be completely dissolved in N-methyl pyrrolidone, while ImOPBI-5, ImOPBI-10, and ImOPBI-15 could not be dissolved. The gel content increased with the increase in the cross-linking agent, and the fractions were measured to be 76.1%, 93.4%, and 98.7%, respectively, which further suggested that cross-linking agents were successfully introduced to the OPBI chains.

Moreover, the Br contents and conversion rates of the membranes before and after grafting with 1-methylimidazole were characterized by X-ray fluorescence spectroscopy to further verify the introduction of Br-HPP and Im to the OPBI membranes. As shown in Table 1, the contents of Br increased with the increase in the cross-linking agents, ranging from 2.09% to 6.72%. After immersion in 1-methylimidazole and NaOH solutions, the Br content in the ImOPBI membrane decreased. As seen by combining these findings with the FTIR spectra, the imidazolium groups were successfully introduced into the OPBI membranes. Based on the Br content before and after immersion in 1-methylimidazole, the conversion rate could be calculated to range from 87.6% to 90.3%.

### 3.2. Thermal and Oxidative Stability

**Figure 2:**
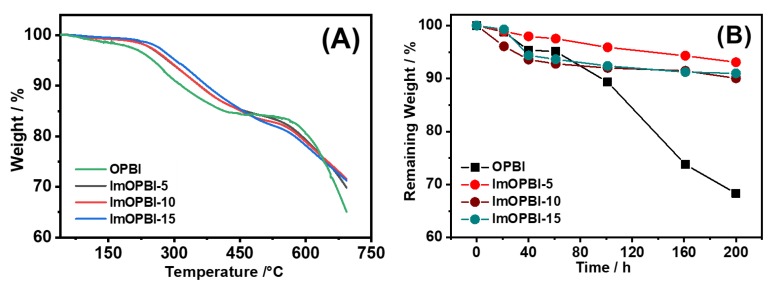
(**A**) Thermogravimetric analysis (TGA) and (**B**) oxidative stability of OPBI and ImOPBI-x membranes.

The thermal stability of the ImOPBI-x membranes was evaluated by thermogravimetric analysis (TGA) under a nitrogen flow, and T_5%_ was defined as the temperature at which the weight loss reached 5%. As shown in Figure 2A, the ImOPBI-x membranes displayed higher thermal stability than the linear OPBI membrane. For the ImOPBI-x membranes, the T_5%_ of ImOPBI-5, ImOPBI-10, and ImOPBI-15 were 287, 287, and 300 °C, respectively. The first weight loss was from 235 to 343 °C, and we ascribe it to the degradation of methyl and methylene groups. The weight loss between 450 and 600 °C corresponded to the decomposition of the main polymer chain. The TGA results indicate that all membranes exhibited excellent thermal stability, which is suitable for applications in HT-PEMFCs.

The ex situ oxidation stability of the membrane material was evaluated by a Fenton test, which exposed membranes of the same size to 3 wt% H_2_O_2_ and 4 ppm Fe^2+^ solution at 68 °C for almost 200 h. The remaining weights of the membranes were recorded at different times, as shown in Figure 2B. Within 100 h of the Fenton test, the mass loss of the OPBI film was small. However, after 100 h, the OPBI membrane became brittle and cracked, and the mass loss increased sharply. After 200 h of the Fenton test, only 68.3% of the residual mass of the OPBI film remained. By comparison, the ImOPBI-x membranes did not show obvious damage, and the residual mass remained within 90.0% after the 200 h test. The linear structure of the OPBI membrane makes it vulnerable to attack by peroxide and hydroxyl radicals (·OOH and·OH) formed from the anode free radicals, resulting in a sudden decrease in the molecular weight. For ImOPBI-x membranes, although the alkyl structure of the cross-linking agent is vulnerable to being attacked by the free radicals, the large volumes of the cross-linking structure cause the polymer to form a three-dimensional structure. When ImOPBI-x membranes are attacked by free radicals, breaking some molecular chains will not cause the polymer skeleton to degrade, keeping the membrane morphology almost intact [31]; thus, the mass loss was low even after treatment in Fenton reagent for 200 h. In conclusion, ImOPBI-x films had good oxidation stability. However, with the increase of the cross-linking agents, the oxidation stability decreased due to the crosslinkers’ many methyl and methylene groups, which are easily oxidized. Thus, there was a balance between them, and here ImOPBI-5 displayed the best performance.

### 3.3. Phosphoric Acid Uptake and Retention Property

The PA uptake is relevant to the structure of the polymers. Generally, branching polymers with a large free volume can have a larger amount of PA sorption ability than linear polymers under the same conditions. The introduction of imidazolium groups can also lead to enhanced PA uptake by forming ion pairs with phosphate ions. However, cross-linking agents cause polymer molecular chains to align closely, which will decrease the PA uptake. Therefore, when the cross-linking degree is not high, more phosphoric acid can be doped in the membranes. As shown in Table 2, the PA uptake of the OPBI film was 211.6%. When OPBI contained the hyperbranched cross-linked agents decorated with imidazolium groups, the PA uptake of the membranes increased, and ImOPBI-5 exhibited a PA uptake of 220.8%. However, when the content of cross-linking agents was further increased (10%–15%), the PA uptake decreased due to the cross-linking behavior, which made the membrane more compact and less PA could be accommodated among polymer chains.

**Figure 3:**
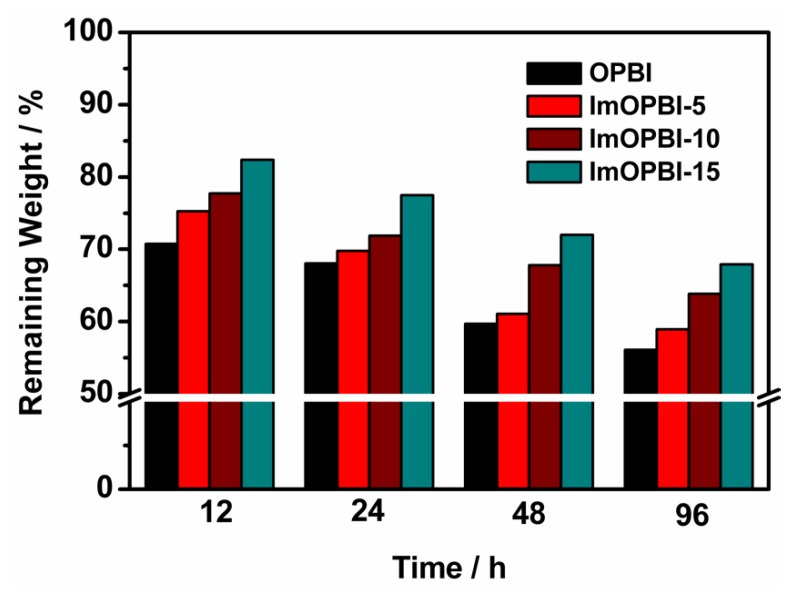
PA retention ability of membranes.

During the operation of HT-PEMFCs, water is continuously generated in the cathode, which will remove the phosphoric acid doped by the electrolyte membrane, leading to a decreased performance after long-term operation. To simulate the phosphoric acid loss process of the membranes, the PA-doped membranes were placed at 80 °C and 40% relative humidity (RH), through which the PA loss by water condensation could be observed [24]. Phosphoric acid retention of the membranes is shown in Figure 3. After 96 h of testing, the residual phosphoric acid content in the OPBI membrane was only 56.1%, which is lower than in the ImOPBI-x membranes, ranging from 58.9% to 67.9%. More phosphoric acid could be retained in ImOPBI-x membranes because the cross-linking agents made the polymer form a three-dimensional network structure, increasing the resistance to phosphoric acid. In addition, hyperbranched structures with tiny cavities could help to absorb PA and inhibit its migration. Furthermore, the introduced imidazolium salt structures could form strong interactions with PA molecules by forming Im^+^···H_2_PO_4_·(H_3_PO_4_)_n_ pairs, thus reducing the phosphoric acid loss [34]. Therefore, the introduction of hyperbranched cross-linked agents decorated with imidazolium groups could effectively improve the phosphoric acid retention ability of the membrane materials.

### 3.4. Proton Conductivity

**Figure 4:**
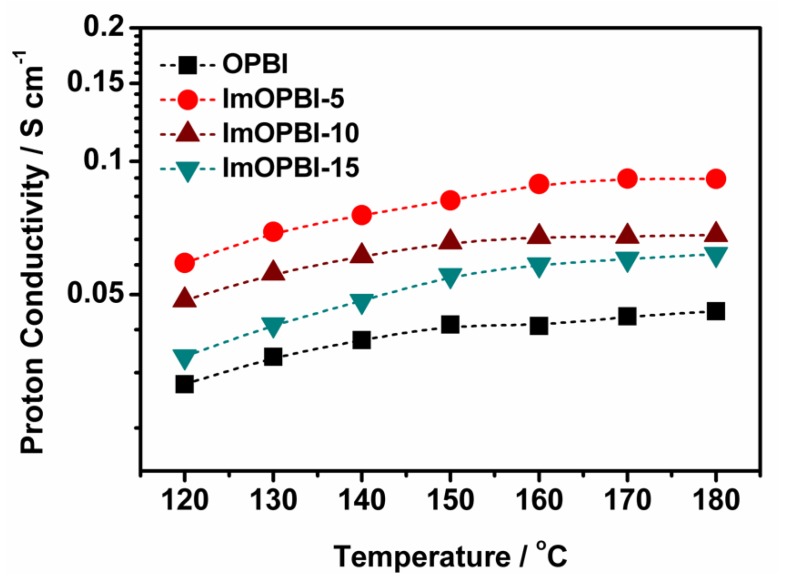
Proton conductivity of as-doped membranes.

Proton conductivity is one of the most important factors to assess the performance of PEMs. The proton conductivity of ImOPBI-x membranes was evaluated from 120 to 180 °C under anhydrous conditions, and OPBI was used as the control. As shown in Figure 4, the proton conductivity of membranes based on OPBI was higher than that of the PBI membrane, all of which increased with increasing temperature. When the test temperature was higher than 160 °C, the proton conductivity of all membrane materials increased slowly due to the condensation and dehydration of phosphoric acid at higher temperatures. The proton conductivity of OPBI reached 0.043 S cm^−1^ at 160 °C (Table 2). For ImOPBI-5, the proton conductivity reached 0.089 S cm^−1^ at 160 °C due to the increase in the phosphoric acid doping rate, which was higher or comparable to that based on PBI membranes [35,36,37]. With the increase in the cross-linking degree, although the phosphoric acid content of ImOPBI-10 and ImOPBI-15 decreased, more imidazolium groups were introduced into the membranes, which not only acted as a Bronsted base but also promoted phosphoric acid ionization to form ion pairs with phosphoric acid (Im^+^···H_2_PO_4_·(H_3_PO_4_)_n_), providing transfer sites for proton transfer. Therefore, both ImOPBI-10 and ImOPBI-15 exhibited good proton conductivity, with values of 0.067 S cm^−1^ and 0.058 S cm^−1^ at 160 °C, respectively (Table 2). Compared to the proton conductivity of QOPBI-x decorated with trimethylammonium groups, that of ImOPBI-x with a higher PA content was higher under the same conditions because imidazole has a conjugated ring, which can delocalize the cation of the imidazolium to the whole ring to contact more phosphoric acid molecules. In addition, the large volume of imidazolium groups also facilitated the accommodation of more PA molecules [38].

### 3.5. Mechanical Properties

**Figure 5:**
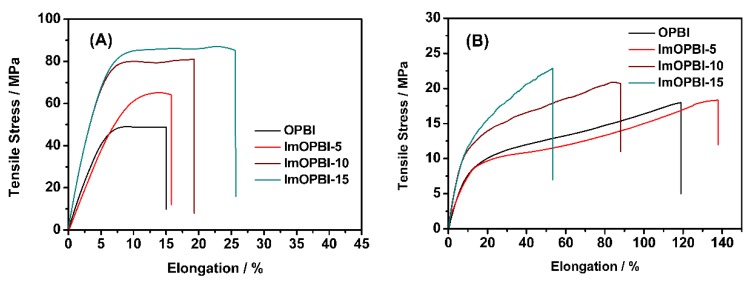
(**A**) Stress–strain curves of dry membranes. (**B**) Stress–strain curves of doped membranes.

Polymer-based HT-PEMs must have excellent mechanical properties; however, high PA adsorption will decrease the mechanical strength of PBI membranes because PA reduces the interaction between molecular chains. The mechanical performance of the membranes before and after PA doping was measured (Figure 5), and the specific values are listed in Table 2. Before doping, the tensile strength of OPBI was 49.3 MPa. The ImOPBI-x had higher tensile strength, ranging from 65.5 to 87.2 MPa. This is because hyperbranched cross-linkers connected the molecular chains of the polymers, enhancing the relationship among chains. Compared with the linear OPBI membrane, the breaking at elongation of the ImOPBI-x membranes increased significantly, which may be related to the existence of a large number of methylene groups in the structural units of the hyperbranched cross-linkers.

After doping with phosphoric acid, the tensile strength of the polymer membranes decreased, while the breaking at elongation increased due to the plasticizing effect of PA. The tensile strength of ImOPBI-x doped with PA was higher than that of the linear OPBI membrane under the same conditions. In addition, the tensile stress increased when the degree of cross-linking increased, and ImOPBI-15 displayed the highest tensile strength (21.9 MPa), which meets the requirements of fuel cells.

### 3.6. Fuel Cell Performance and Durability

**Figure 6:**
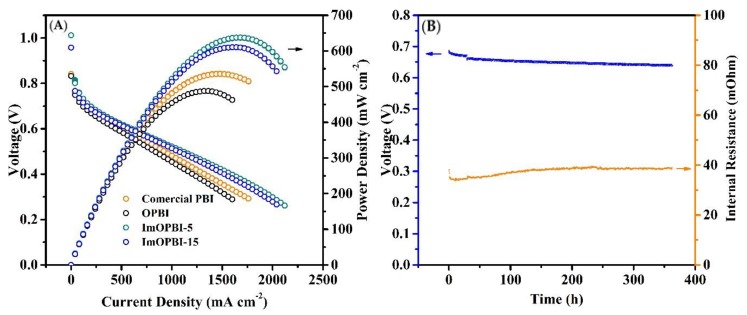
(**A**) Single-cell performance based on commercial PBI, OPBI, ImOPBI-5, and ImOPBI-15 membranes. (**B**) Durability of ImOPBI-5 in terms of the cell voltage at 160 °C and a constant current load of 200 mA cm^−2^.

The single cell performances of OPBI, ImOPBI-5, and ImOPBI-15 were tested, and the PBI membrane was used as the control. Hydrogen and oxygen were injected into the anode and cathode, respectively, and their power density and internal resistances were tested. As shown in Figure 6A, the power density of the fuel cell based on ImOPBI-5 and ImOPBI-15 reached 638 mW cm^−2^ and 610 mW cm^−2^ at 160 °C, respectively, which was higher than those based on OPBI and PBI. Since the ImOPBI-5 film had the highest power density, the durability in terms of the cell voltage at 160 °C was evaluated (Figure 6B). The data showed almost no voltage degradation after the battery was discharged continuously for 390 h, indicating the good chemical stability and PA retention ability of the ImOPBI-5.

## 4. Conclusions

In conclusion, polybenzimidazole membranes containing different hyperbranched cross-linkers decorated with imidazolium groups (ImOPBI-x) were prepared and their performance as HT-PEMFCs was evaluated. The results indicate that the imidazolium groups could accommodate more PA molecules and stabilize them by delocalization and hydrogen bond formation, due to their conjugated rings. PEMs based on ImOPBI-x had excellent oxidation stability, high proton conductivity, and acceptable tensile strength, meeting the requirements of HT-PEMFCs. The power density of the fuel cell based on ImOPBI-5 reached 638 mW cm^−2^ at 160 °C under anhydrous hydrogen/oxygen.

## Figures and Tables

**Table 1:** The Br content and conversion rate of membranes before and after grafting 1-methylimidazole and NaOH.

	Br Content before Soaking/%	Br Content after Soaking/%	Conversion Rate/%
ImOPBI-5	2.09	0.26	87.6
ImOPBI-10	4.14	0.40	90.3
ImOPBI-15	6.72	0.67	90.0

**Table 2:** Gel fraction, thickness, PA uptake, proton conductivity, and tensile stress of membranes.

Sample	PA Uptake/%	Proton Conductivity at 160 °C/S cm^−1^	Tensile Stress/MPa	
			Undoped	Doped
OPBI	211.6	0.043	49.3	18.0
ImOPBI-5	220.8	0.089	65.5	18.3
ImOPBI-10	191.6	0.067	81.1	21.0
ImOPBI-15	173.0	0.058	87.2	22.9

## Supplementary Materials

The materials, the preparation of OPBI, Br-HPP, and testing methods are available online at https://www.mdpi.com/2073-4360/12/3/515/s1.
