# Supplementary material for: Synthesis and Properties of Phosphoric-Acid-Doped Polybenzimidazole with Hyperbranched Cross-Linkers Decorated with Imidazolium Groups as High-Temperature Proton Exchange Membranes
Source: Polymers (Basel). 2020 Feb 27;12(3):515. doi: 10.3390/polym12030515 (PMC7182959; doi:10.3390/polym12030515)
Supplement: Supplementary file 1 [file polymers-12-00515-s001.pdf]

Supporting Information

Synthesis and properties of phosphoric-acid-doped polybenzimidazole with hyperbranched cross-linkers decorated with imidazolium groups as high-temperature proton exchange membranes

Chunmei Gao [1], Meishao Hu [2], Li Wang [2], Lei Wang [2,]*

[1]College of Chemistry and Environmental Engineering, Shenzhen University, Shenzhen 518060, China

[2]Shenzhen Key Laboratory of Polymer Science and Technology, College of Materials Science and Engineering, Shenzhen University, Shenzhen 518060, China

**Correspondence: wl@szu.edu.cn*

1. Materials

1,2-Dichloromethane (DCM), 4-methylbenzyl chloride and $SnCl_4$ were purchased from Aladdin and used as received except DCM. DCM was dried with calcium hydride and distilled before use. N-bromosuccinimide (NBS) was purchased from TCI (Shanghai) Development Co., Ltd. Sodium hydroxide, phosphoric acid, ether and acetone were purchased from Tianjin Yongda Chemical Reagent Company. Carbon tetrachloride and NMP were dried over by 4-Å molecular sieves before use. The other agents were purchased from the Energy Chemical company.

2. Preparation of OPBI and Br-HPP

OPBI, Br-HPP, and the cross-linked OPBI-x membranes were synthesized according to the method reported in our previous paper [1].

3. Gel Fraction

The prepared membranes (1 × 3 cm^2) were immersed into NMP at 80 °C for 24 h. The undissolved membranes were subsequently collected, washed with methanol and deionized water, and then dried at 120 °C for 24 h under vacuum. The gel fraction of the membranes was characterized according to the formula below:

Gel fraction (%) = $W_1 / W_0 \times 100\%$

Where W_0 and W_1 are the quality of sample membrane before and after immersion in NMP, respectively.

4. Phosphoric Acid Uptake

The phosphoric acid doping level was calculated by measuring the change of weight of dry membrane samples before and after being immersed in 85% phosphoric acid according to reference [2].

$$\text{PA uptake (\%)} = \frac{W_{Doped\ membrane} - W_{Dry\ membrane}}{W_{Dry\ membrane}} \times 100\ \% \qquad (1)$$

Where $W_{Dry\ membrane}$ represents the weight of the dry membrane before being immersed in phosphoric acid and $W_{Doped\ membrane}$ represents the weight of the dry membrane after being immersed in 85% phosphoric acid.

5. Phosphoric Acid Retention

After the membranes were cut into squares (2 × 3 cm^2), they were placed in an environmental chamber at 80 °C and 40% humidity for different amounts of time, and then the weight of membranes was tested.

6. Proton Conductivity

The proton conductivity of the PA-doped membrane (1 × 4 cm^2) was measured under anhydrous conditions on a CHI660e electrochemical workstation using a two-electrode AC impedance method over the frequency range of 1 to 10^5 Hz at temperatures from 120 to 180 °C. The proton conductivities were calculated by the following formula:

$$\sigma\ (\mathrm{S\ cm^{-1}}) = \frac{L}{R \times A} \qquad (4)$$

where σ is the proton conductivity, *L* is the distance between the two electrodes, *R* is the membrane resistance derived from the low intersection of the high-frequency semicircle on a complex impedance plane with the real (Z) axis, and *A* is the cross-sectional area of the testing sample.

7. Mechanical Properties

The membrane samples were immersed in 85% PA for 24 h and then dried at 120 °C for 24 h. The mechanical properties of the samples were measured using an Instron model 1185 analyzer under ambient conditions at a test speed of 10 mm min^{-1}. The samples were cut into 15 × 4 mm^2 dumbbell shapes using a special cutter.

8. Oxidative Stability

The membrane was submerged in Fenton's solution (4 ppm Fe^{2+} in 3 wt % H_2O_2) at 68 °C for a certain time, washed with deionized water, and dried at 120 °C for 12 h before weighing [3,4].

9. Fuel Cell

Dry hydrogen and oxygen were applied to the fuel cell at flow rates of 80 and 160 mL min^{-1}, respectively. The area of each of the membrane electrode assemblies (MEAs) was 2.25 × 2.25 cm^2. The electrodes, 1.0 mg cm^{-2} Pt/C for the anode and the cathode, were purchased from Hensen Company. The active area of each of the MEAs was 5 cm^2. Polarization curves were obtained using current-step potentiometry.

References

[1] M. Hu, T. Li, S. Neelakandan, L. Wang, Y. Chen, Cross-linked polybenzimidazoles containing hyperbranched cross-linkers and quaternary ammoniums as high-temperature proton exchange membranes: Enhanced stability and conductivity, J. Membr. Sci., 593 (2020) 117435.

[2] X. Tian, S. Wang, J. Li, F. Liu, X. Wang, H. Chen, D. Wang, H. Ni, Z. Wang, Benzimidazole grafted polybenzimidazole cross-linked membranes with excellent PA stability for high-temperature proton exchange membrane applications, Appl. Surf. Sci., 465 (2019) 332-339.

[3] J.S. Yang, Q.F. Li, L.N. Cleemann, C.X. Xu, J.O. Jensen, C. Pan, N.J. Bjerrum, R.H. He, Synthesis and properties of poly(aryl sulfone benzimidazole) and its

copolymers for high temperature membrane electrolytes for fuel cells, J Mater Chem, 22 (2012) 11185-11195.

[4] J.S. Yang, Q.F. Li, L.N. Cleemann, J.O. Jensen, C. Pan, N.J. Bjerrum, R.H. He, Crosslinked hexafluoropropylidene polybenzimidazole membranes with chloromethyl polysulfone for fuel cell applications, Adv. Energy Mater., 3 (2013) 622-630.
